# A novel method for repeated cerebrospinal fluid sampling and long-term monitoring of intracranial pressure in rats

**DOI:** 10.3389/fnins.2023.1110537

**Published:** 2023-02-17

**Authors:** Guangshan Hao, Qia Zhang, Weiyu Chen, Jun Mo

**Affiliations:** ^1^Department of Neurosurgery, The Fourth Affiliated Hospital, School of Medicine, Zhejiang University, Yiwu, Zhejiang, China; ^2^Department of Neurosurgery, Liaocheng People’s Hospital, Liaocheng, Shandong, China; ^3^International Institutes of Medicine, The Fourth Affiliated Hospital Zhejiang University School of Medicine, Yiwu, Zhejiang, China

**Keywords:** cisterna magna, cannulation implantation, intracranial pressure monitoring, cerebrospinal fluid collection, neuroscience research

## Abstract

Cannulation implantation into the cisterna magna is an important procedure in cerebrospinal fluid (CSF) sampling and intracranial pressure (ICP) monitoring. The disadvantages of existing techniques include the risk of brain damage, compromised muscle mobility, and the complexity of the procedures. In the present study, the authors describe a modified, simple, and reliable procedure for long-term cannulation implantation into the cisterna magna in rats. The device consists of four parts: the puncture segment, the connection segment, the fixing segment, and the external segment. Intraoperative ICP monitoring and post-operative computed tomography (CT) scans were performed, which confirmed the accuracy and safety of this method. There were no limitations on the daily activities of the rats when long-term drainage was carried out for 1 week. This new technique offers an improved method of cannulation and will be a potentially useful method for CSF sampling and ICP monitoring in neuroscience research.

## 1. Introduction

Cerebrospinal fluid (CSF) is an ultrafiltrate of plasma mainly produced by the choroid plexuses (Di Terlizzi and Platt, 2006). It plays an important role in cushioning the brain and spinal cord and maintaining intracranial pressure (ICP), and also functions as a medium to remove metabolic waste as well as to transport neuromodulators and neurotransmitters (Shen et al., 2004; Spector et al., 2015; Ramos et al., 2019). CSF furnishes a window to track pathophysiological and biochemical changes in the central nervous system (CNS), and varies tools including CSF sampling and ICP monitoring have been commonly used in neuroscience research (Lundberg, 1960; Bouman and Van Wimersma Greidanus, 1979; Barth et al., 1992; Pegg et al., 2010; Shapiro et al., 2012). However, long-term cannulation for repeated CSF acquisition and ICP monitoring remains challenging due to fixation difficulties, risk of brain injury, and impacts of the daily activities of animals.

Given the importance of CSF, several manipulation techniques have been described previously to study it in preclinical research, including the lumbar puncture (Kusaka et al., 2004), percutaneous cisterna magna puncture (Mahat et al., 2012), and cannulation implantation into the cisterna magna (Xavier et al., 2018) or lateral ventricle (Uldall et al., 2014). Among those, cisterna magna cannulation implantation has become a research hotspot in recent years due to its reliability and non-invasiveness to brain tissue. Long-term cannulation implantation in the cisterna magna has been performed mainly *via* two methods: the transcranial approach and the open surgical approach to the cisterna magna. The former implants a catheter into the cisterna magna *via* a surgical trans-occipital bone approach that introduces a catheter into the cisterna magna through the long, blind way between the occipital bone and the cerebellum (Shapiro et al., 2012). However, this method has disadvantages, such as poor controllability of the position of the catheter and possible damage to the brain and integrity of the subarachnoid space. Moreover, drilling into the skull is complicated to some extent, disrupting the integrity of the skull, and potentially leading to the disruption of the intracranial environment. The latter approach is to separate the muscles at the back of the neck and puncture the atlantooccipital membrane under direct vision. Two methods have been described to fix the catheter implanted in the cisterna magna. One method reported by Xavier et al. (2018) is to fix the catheter with a mixture of dental cement glue, which may be unstable and affect the activity of local muscles. Another by Barth et al. (1992) is a complicated fixed system that requires drilling into the skull, and the complex cannulation system has a certain mass effect that can affect the activity of local muscles. Thus, a simple, injury-free and reliable cannulation system and implantation method were needed for further research.

In this study, we describe a modified, simple, and reliable method for long-term cannulation implantation into the cisterna magna in rats, which would facilitate repeated CSF sampling and long-term monitoring of ICP in neuroscience research.

## 2. Materials and methods

### 2.1. Animals

We used male Sprague–Dawley (SD) rats weighing 280–300 g (SLAC Laboratory Animal Co., Ltd., Shanghai, China) in this study. We kept the rats in a 12-h day/night cycle at 22 ± 1°C and 50 ± 10% humidity. The rats had free access to water and food. All animal experiments were approved by the Institutional Animal Care and Use Committee of Zhejiang University.

### 2.2. Study design

A total of 15 rats were divided into two groups. Group A (*n* = 9): rats underwent surgery with cannulation implantation. Group B (*n* = 6): rats underwent a sham operation and no cannulation was implanted. In group A, cannulation patency inspection, ICP recording, body weight measurements were taken on day 0 (the day of surgery), day 3, and day 7. In group B, body weight measurements were taken on day 0, day 3, and day 7.

### 2.3. Cannulation design

**FIGURE 1:**
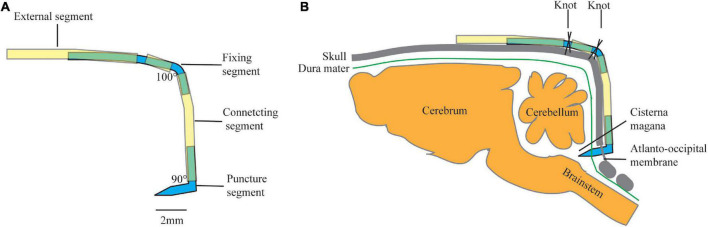
Schematic of cannulation design: **(A)** diagram of a constructed cannula, and **(B)** schematic drawing of annulation placement in rat. Yellow tube: polyethylene (PE) tube (PE50), Blue tube: modified 18 G syringe needle.

The design of the cannulation consists of four parts: (1) a puncture segment with a fixing angle and length to ensure the direction and depth of the puncture, (2) a flexible and adjustable connecting segment to ensure the stability of the puncture segment, (3) a fixing segment with an angle and a polyethylene (PE) tube to ensure that the entire device is firmly fixed to the skull, and (4) an external segment for CSF sampling or connecting ICP monitor (Figure 1).

#### 2.3.1. Puncture segment

The puncture segment is made of a modified 18 G syringe needle (outer diameter: 0.7 mm) with an angle of 90° and a length of 2-mm at each end. One of the ends with a beveled tip was used to puncture the atlantooccipital membrane, and the other end was connected to a PE tube (PE 50).

#### 2.3.2. Connecting segment

The connecting segment was a 5-mm long PE tube (PE 50). One end was connected to the puncture segment, while the other end was connected to the fixing segment.

#### 2.3.3. Fixing segment

The fixing segment is made of a modified 18 G syringe needle (outer diameter: 0.7 mm) with an angle of 100°. The proximal end was 2 mm in length and is connected to the connecting segment. In the middle, the fixing segment was covered with a 1-mm PE pipe, fixed by a small amount of glue. The distal end was 4 mm in length which was connected to the external segment.

#### 2.3.4. External segment

The external segment was a 6-mm long PE tube. The proximal end is connected to the fixing segment, while the distal segment is connected to a syringe pump for CSF sampling or a transducer for ICP monitoring. It could also be blocked temporarily for long-term retention.

### 2.4. Surgical procedures

#### 2.4.1. Anesthesia

The animals were anesthetized *via* an intraperitoneal injection of chloral hydrate (10%, 300 mg/kg). The proper anesthesia level was achieved within 2–3 min and verified by the lack of a flick response when pinching the tail tip with forceps.

#### 2.4.2. Position and incision

Each animal was mounted in a stereotaxic apparatus with the nose slightly facing downward. The back of the head and neck was shaved, and then skin sterilization was performed with ethyl alcohol (70%). A 3-cm incision was made at the midline with the midpoint between the ears.

#### 2.4.3. Fascia and muscle dissection

**FIGURE 2:**
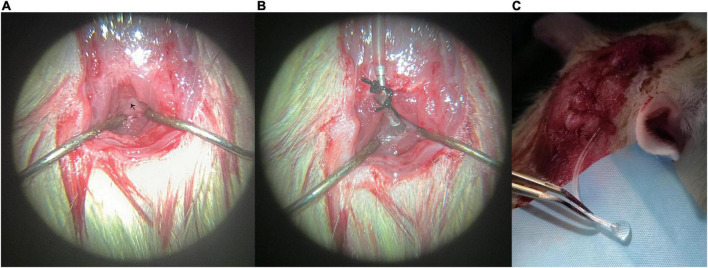
Representative photos of the modified cannulation implantation: **(A)** fascia and superficial muscles were bluntly separated at the midline, and the atlantooccipital membrane was exposed, **(B)** cannulation was implanted into the cisterna magna, and **(C)** clear cerebrospinal fluid (CSF) can be seen flowing out of the external segment (PE tube). CSF, cerebrospinal fluid. Black arrow marks the atlantooccipital membrane.

This step was similar to that reported previously (Pegg et al., 2010; Barthel et al., 2021). Briefly, the fascia and superficial muscles were bluntly separated at the midline. The two layers of muscles below were exposed and separated from the midline by blunt dissection to avoid bleeding. The muscles were retracted laterally by employing two self-made retractors, creating a gap in the midline, and exposing the atlantooccipital membrane. Any bleeding was stopped by cotton swabs or cauterization. One prepared thread was pre-sewn to the tendon at the external occipital crest for fixation, and a second thread was pre-sewn to the tissue 1 mm distally away. The two threads were knotted at the fixed segment to secure the whole device (Figure 2A).

#### 2.4.4. Cannulation implantation

The cannulation was first connected to a 1-ml syringe and filled with artificial CSF (aCSF) avoiding bubbles in the tube. Then the beveled tip was inserted into the atlantooccipital membrane at the midline just below the occipital bone. A little cyanoacrylate glue was applied to seal the puncture site avoiding CSF leak, then the whole cannulation was placed close to the occipital bone and fixed with the two preset threads on fixing segment. The cannulation was kept in place with the help of the two knots. Meanwhile, the connecting segment can provide tension to the puncture segment, and keep the cannulation tip in place without moving or falling out (Figure 2B). Recipe of aCSF: aCSF was prepared as the combination of solution A (NaCl 8.66 g, KCl 0.224 g, CaCl_2_⋅2H_2_O 0.206 g, and MgCl_2_⋅6H_2_O 0.163 g dissolved in 500 ml pyrogen-free, sterile water) and B (Na_2_HPO_4_⋅7H_2_O 0.214 g, NaH_2_PO_4_⋅H_2_O 0.027 g dissolved in 500 ml pyrogen-free, sterile water) in a 1:1 ratio.

#### 2.4.5. Closure

The muscles were restored in place, and the skin was sutured and sterilized.

### 2.5. ICP recording and Cannulation patency inspection

Under proper anesthesia *via* an intraperitoneal injection of chloral hydrate (10%, 300 mg/kg), ICP recording was performed by connecting the external segment to ICP monitoring device (MP150, BIOPAC Systems, Inc.). ICP was continuously monitored for 10 min, data of the last 5 min was analyzed with frequency set at 1 Hz and the average was used for further analysis.

Cannulation patency was checked on day 0, day 3, and day 7. Cannulation patency was confirmed when clear CSF was seen gushing out of the external PE tube (Figure 2C).

### 2.6. CT scan

Following anesthesia with chloral hydrate (10%, 300 mg/kg, i.p.), rats underwent CT scans of the head and neck (SOMATOM Definition AS + 128-slice spiral CT; Siemens Healthineers, Forchheim, Germany). The CT scan conditions were 1.0 mm slice thickness, 1.0 mm interval, 1.0 pitch, 120 kV tube voltage, 60 mA tube current, 512 × 512 matrix, and FOV 9.6 cm, 50 s scanning.

### 2.7. Statistical analysis

GraphPad Prism 9 (GraphPad, San Diego, CA, USA) was used for statistical analysis. Shapiro–Wilk normality test was applied to test for normal distribution. Values are presented as percentage of positive results (%) or mean ± standard deviation (SD). Differences of body weight gains between two groups were analyzed using two-sided Student *t* test; categorical data were analyzed using Chi-square test. A *p*-value less than 0.05 was considered as statistically significant.

## 3. Results

### 3.1. General information

No rat was dead after operation in both groups. In cannulation implantation group (Group A), all the cannulations were successfully implanted. One rat (11.1%) was found skin incision infection on day 2 and was ruled out. Cannulation in one rat (11.1%) was found blocked on day 3 and was ruled out from further analysis. Cannulations in the other seven rats (77.8%) were all found to be patent on day 0, day 3, and day 7 (Table 1). CSF was not collected in the present study. Post-operative autopsy found no significant macroscopic damage to brainstem and cerebellum (data not shown).

**TABLE 1:** Outcomes comparison between Group A and Group B.

	Mortality rate (%)	Infection rate (%)	Cannulation patency rate (%)	Body weight gain (g)	
				**Day 3**	**Day 7**
Group A (*n* = 9)	0	11.1	77.8	6.5 ± 2.2	23.0 ± 6.3
Group B (*n* = 6)	0	0	NA	6.6 ± 3.7	25.0 ± 5.9
*p*-value	>0.05	>0.05	NA	>0.05	>0.05

Values represent percentage of positive results (%) or mean ± standard deviation. Mortality rate, infection rate, and cannulation patency rate were recorded on day 7.

### 3.2. Body weight gain after operation

The body weight gain after operation on the 3rd and 7th post-operative day between the two groups was compared, and it was found that there was no significant difference in weight gain between the two groups at the two time points (Table 1).

### 3.3. CT imaging

**FIGURE 3:**
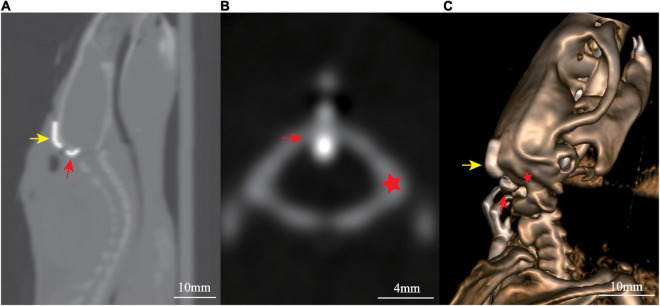
Post-surgical computed tomography (CT) scan showed the localization of cannulation: **(A)** sagittal view, **(B)** axial view, and **(C)** three-dimensional reconstruction. Yellow arrow marks the fixing segment, while red arrow marks the puncture segment. The red star marks the occipital bone.

Post-operative CT scan on the 7th day showed the cannulation system was tightly attached to the occipital bone and the tip of the tube located in the cisterna magna (Figure 3).

### 3.4. ICP recording

**FIGURE 4:**
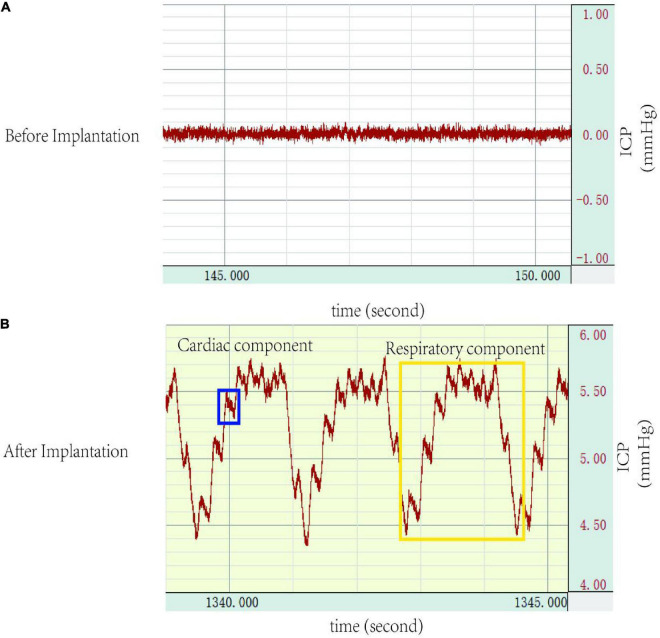
Intracranial pressure (ICP) waveforms: **(A)** no waveforms were demonstrated before cannulation implantation into the cisterna magna, **(B)** a representative ICP trace after successful cannulation implantation into the cisterna magna detailing ICP waveforms, which shows a cardiac waveform (blue), and a respiratory waveform (yellow). ICP, intracranial pressure.

After connecting the ICP monitoring device (MP150, BIOPAC Systems, Inc.), normal ICP waveforms with cardiac and respiratory components were noted (Figure 4). In group A, no significant difference was found in ICP values on day 0, day 3, and day 7 (5.09 ± 0.22 mmHg vs. 5.12 ± 0.20 mmHg vs. 5.11 ± 0.40 mmHg, *p* > 0.05).

## 4. Discussions

Cisterna magna cannulation implantation has become the main method of CSF acquisition and ICP monitoring in recent years because of its non-invasive to brain tissue and reliability of operation. However, long-term cannulation implantation into cisterna magna remains challenging due to fixation difficulties, risk of brainstem injury, and affecting the daily activities of animals. In the present study, we designed a new cannulation system and described a modified procedure for long-term implantation in the cisterna magna of rats.

There are two main methods reported for long-term cannulation implantation in the cisterna magna: the trans-occipital approach and the trans-atlantooccipital membrane approach. In the trans-occipital approach, a catheter was introduced into the cisterna magna through a long and blind way between the occipital bone and the cerebellum (Shapiro et al., 2012). The advantage of this approach is that the puncture tube is stable, but the disadvantages are also obvious, including uncertainty about the site of puncture and the possibility of brain tissue damage. In the trans-atlantooccipital approach, by separating the neck muscles, the atlantooccipital membrane can be punctured under direct vision, which greatly improves the accuracy of puncture and avoids brain tissue damage. However, fixation is relatively difficult with this method. There are two methods have been described to secure catheter implanted in the cisterna magna. Uldall et al. (2014) and Xavier et al. (2018) applied a mixture of dental cement glue topically to fix the catheter, which can affect the movement of the neck muscles, and cause instability of the catheter. Barth et al. (1992) fixed the puncture tube by drilling holes in the skull, but the procedure is complex and also affects the movement of neck muscles.

The present modified cannulation system and procedure have several advantages compared with those previously reported. First, with the 90° design of the puncture segment, the puncture can be performed under direct vision. It is easy to control the puncture direction and depth, avoiding blood contamination, and cerebellum damage. Second, the connecting segment use a flexible PE tube, which not only applies pressure to the puncture segment to ensure its position, but also keeps the entire device closely attach to the skull for stable fixation. Third, by adjusting the overlapping length of the connecting and fixing segments, it can be individually adapted to rats of different sizes. Moreover, the cannulation system has little impact on the movements of the neck muscles, and there is no effect on the daily activities of the animal. There was no significant difference in weight gain between the cannulation implantation group and sham group, suggesting that the modified cannulation implantation did not affect the feeding and water intake of the animals. The reliability of this system and procedure has been confirmed by post-operative CT scan (Figure 3).

Cerebrospinal fluid (CSF) is in direct contact with extracellular space and vessels, and functions as a medium to remove metabolic waste as well as to transport neuromodulators and neurotransmitters. The alterations in CSF can reflect pathophysiological changes of the CNS, the analysis of which has become an invaluable tool in neuroscience research. Several techniques have been described previously to study CSF in preclinical research, including the lumbar puncture (Kusaka et al., 2004), the percutaneous cisterna magna puncture (Mahat et al., 2012). With cannulation implantation, repeated CSF sampling can be performed, which can better reflect temporal biological changes in the CNS and minimize the effects of variability between animals and reduce animal use (Uldall et al., 2014; Xavier et al., 2018). In this study, the modified cannulation system made the procedure simple, safe, and reliable, and we could still get clear CSF 7 days after cannulation implantation without impacts on the daily activities of the animals.

Intracranial pressure (ICP) monitoring has been commonly used as a standard method in research of neurological diseases. Various ICP monitoring methods have been described, which can be divided into lateral ventricle, cerebral parenchymal, cisterna magna, and epidural or subdural spaces according to the monitoring site, each with its advantages and disadvantages. Intraventricular monitoring was first developed by Lundberg (1960) and considered as the gold standard for ICP monitoring. However, the ICP values vary significantly (4–47 mmHg) when using the same method and system, due to different ICP reference planes (Uldall et al., 2014). Moreover, the procedure is complex and requires a stereotactic system, often associated with the risk of brain injury and intracerebral haemorrhage. The cerebral parenchymal monitoring requires a particular and expensive transducer system, and also leads to regional brain damage. In addition, the pressure probe cannot sense pressure correctly due to local brain damage, and the ICP cannot be accurately tracked during repeat or long-term monitoring. Other studies have also monitored ICP in the subdural or epidural space, but the low accuracy limits the application. Cisterna magna monitoring is simple, reliable, and without brain injury, and has been widely used in research (Barth et al., 1992; Conzen et al., 2019). In the present study, the ICP waveform that showed both the respiratory and cardiac components also suggested the reliability of this system and method (Figure 4). Moreover, the stability of ICP values also confirmed the reliability of the device for ICP measurement.

Limitations of the present study and suggestions for further study: first of all, each section of the cannulation needs to be self-made, which has some difficulties. Secondly, body weight changes are a measure of general health but are not a great measure of post-surgical mobility, the present study lacks a scale of neck muscle activity examination, and needs to be further refined in the future experiment. Third, there is a lack of randomized controlled trials comparing this study with previous methods. Fourth, we only assessed the cannulation patency for 7 days, and it is unclear whether the cannulation system can be left for a longer time. Finally, CSF was not collected and analyzed for metabolite content, red blood cell content, and infection in the present study.

## 5. Conclusion

The present study described a modified, simple, and reliable method for the implantation of a cannula in the cisterna magna, which can be useful for repeated CSF sampling and long-term ICP monitoring in neuroscience research.

## Data availability statement

The raw data supporting the conclusions of this article will be made available by the authors, without undue reservation.

## Ethics statement

The animal study was reviewed and approved by Institutional Animal Care and Use Committee of Zhejiang University.

## Author contributions

GH: conceptualization, investigation, and writing – original draft preparation. QZ and WC: review and editing. JM: writing – review and editing, investigation, supervision, and funding acquisition. All authors have read and agreed to the published version of the manuscript.
